# Effects of the Adult Attachment Projective Picture System on Oxytocin and Cortisol Blood Levels in Mothers

**DOI:** 10.3389/fnhum.2016.00627

**Published:** 2016-12-08

**Authors:** Sabrina Krause, Dan Pokorny, Katharina Schury, Cornelia Doyen-Waldecker, Anna-Lena Hulbert, Alexander Karabatsiakis, Iris-Tatjana Kolassa, Harald Gündel, Christiane Waller, Anna Buchheim

**Affiliations:** ^1^Department of Psychosomatic Medicine and Psychotherapy, Ulm UniversityUlm, Germany; ^2^Clinical and Biological Psychology, Institute of Psychology and Education, Ulm UniversityUlm, Germany; ^3^Institute of Psychology, University of InnsbruckInnsbruck, Austria

**Keywords:** oxytocin, cortisol, attachment representation, stress, adult attachment projective picture system

## Abstract

Oxytocin, a small neuropeptide of nine amino acids, has been characterized as the “hormone of affiliation” and is stimulated, for instance, in mothers when interacting with their offspring. Variations in maternal oxytocin levels were reported to predict differences in the quality of care provided by mothers. In this study, the Adult Attachment Projective Picture System (AAP) as a valid measure to assess attachment representations was used as an activating attachment-related stimulus. We investigated whether the AAP induces a release of oxytocin in mothers with a secure attachment representation and a stress-related cortisol response in mothers with an insecure attachment representation. Therefore, pre-post effects of AAP administration on plasma oxytocin and serum cortisol levels were investigated in *n* = 44 mothers 3 months after parturition. Oxytocin levels increased from pre to post in the significant majority of 73% participants (*p* = 0.004) and cortisol decreased in the significant majority of 73% participants (*p* = 0.004). Interestingly, no association between alterations in oxytocin and cortisol were found; this suggests taking a model of two independent processes into considerations. These results show that the AAP test procedure induces an oxytocin response. Concerning the results within the four AAP representation subgroups, our hypothesis of a particularly strong increase in oxytocin in secure mothers was not confirmed; however, in secure mothers we observed a particularly strong decrease in cortisol. Effect sizes are reported, allowing the replication of results in a larger study with sufficient sample size to draw final conclusions with respect to differences in OT and cortisol alterations depending on attachment representation. When interpreting the results, one should keep in mind that this study investigated lactating mothers. Thus, the generalizability of results is limited and future studies should investigate non-lactating healthy females as well as males and include a control stimulus condition.

## Introduction

### Attachment as a Biobehavioral System

Attachment is a state in which several physiological and behavioral systems are organized in order to provide an individual with a certain sense of security and protection with others (Bowlby, 1969). Bowlby defines these behavioral systems as biological systems that work along with physiological processes. Mental representations of early attachment relationships shape emotional and cognitive information, which affects the attention and memory as well as the emotional reactivity of our central nervous system. In order to maintain organization within the attachment system, emotional reactivity is regulated within the central nervous system (Main et al., 1985; Bretherton, 1993). Following Bowlby (1969) and Ainsworth et al. (1978), the assessment of developmental attachment places an emphasis on activating attachment and “seeing attachment in action” (i.e., in attachment activating contexts; Bowlby, 1969; Ainsworth et al., 1978). Over the last 20 years the psychobiological research on infant and adult attachment has increased dramatically. Attachment patterns have been linked to different ways to regulate emotions. Some researchers even argue that the attachment system is in itself a device of emotion regulation (Vrtička and Vuilleumier, 2012). Studies indicate that, in response to stress, the insecure attachment is related to a heightened adrenocortical activity, heart rate and skin conductance, which is consistent with the hypothesis that attachment insecurity leads to deficits in emotion regulation (Gander and Buchheim, 2015). The majority of neurophysiological studies of adult attachment use self-report measures (Carpenter and Kirkpatrick, 1996; Kim, 2006; Laurent and Powers, 2007; Rochman et al., 2008; Kiss et al., 2011; Dan and Raz, 2012). Only a few studies use narrative interview measures of attachment like the Adult Attachment Interview (AAI) and the Adult Attachment Projective Picture System (AAP; Beijersbergen et al., 2008; Buchheim et al., 2009; Fraedrich et al., 2010; Holland and Roisman, 2010; Behrens et al., 2011). Self-reports assess the subjective evaluation of attachment styles, primarily differentiating between secure and insecure avoidant or anxious attachment (Ravitz et al., 2010). On the other hand, the AAI or AAP are designed to activate an individual’s internal working model of attachment by introducing attachment-related topics (e.g., separation, illness, abuse and death), and they assess attachment representations (e.g., secure, insecure-dismissing, insecure-preoccupied and unresolved trauma) based on the analysis of verbatim transcripts. The feasibility of the AAP measure as a stimulus in an attachment-related neurobiological context has been proven in diverse experimental settings in clinical and nonclinical groups (Buchheim et al., 2006a,b, 2008, 2009, 2012).

### Oxytocin, Attachment and Caregiving

Oxytocin is a neuropeptide of nine aminoacids and is critically involved in both central and peripheral aspects of mammalian attachment and survival. The neuropeptide is mainly produced in the paraventricular and supraoptic nuclei of the hypothalamus. These nuclei project to the posterior pituitary gland. Here, oxytocin is stored coupled with neurophysin I as a precursor complex. Finally, oxytocin is cleaved and is released into the peripheral blood. Oxytocin induces well-described peripheral actions and the oxytocin neurons additionally project to important brain regions manifested in social and maternal behaviors (Braunstein et al., 1980; Insel, 1992; Gimpl and Fahrenholz, 2001; Bielsky and Young, 2004; Strathearn, 2011). Studies on humans and animals have shown that oxytocin mediates anxiolytic effects, and plasma levels are related to the reduction of anxiety and stress (Gimpl and Fahrenholz, 2001; Neumann and Landgraf, 2012). A substantial literature exists underscoring the role of oxytocin in regulating social behaviors (Gimpl and Fahrenholz, 2001; Feldman et al., 2010, 2011; Meyer-Lindenberg et al., 2011). A large number of studies have implicated oxytocin in maternal care (Pedersen et al., 2006), pair bonding (Ross et al., 2009), interpersonal trust (Kosfeld et al., 2005), emotion recognition (Gimpl and Fahrenholz, 2001; Lischke et al., 2012), and empathy (Hurlemann et al., 2010). Furthermore, oxytocin has been characterized as a “hormone of affiliation” and the oxytocinergic system has received attention as a key neural substrate of maternal caregiving, involved in the emergence and maintenance of maternal behaviors (Insel, 1992; Feldman et al., 2010, 2011). Prospective and cross-sectional studies have demonstrated that maternal oxytocin levels are systematically associated with naturally occurring variations in maternal behavior, with high plasma oxytocin levels during pregnancy and postpartum predicting increased maternal behavior in the postpartum months (Gordon et al., 2010; Atzil et al., 2011; Feldman et al., 2011). Interaction with their offspring, in the postpartum period further stimulated oxytocin response in mothers (Feldman et al., 2010, 2011), though significant inter-individual variations have been found in the oxytocin plasma levels (Strathearn, 2011; Strathearn et al., 2012). Additionally oxytocin plays a crucial role in bond formation between children and parents and social reciprocity. Parental oxytocin plasma level and early parental care patterns seemed to be associated with social reciprocity (Feldman et al., 2013; Waller et al., 2015). These natural variations in parental oxytocin responses may predict differences in the quality of maternal/paternal caregiving.

### Oxytocin and Cortisol During Social Stress

Previous research mainly focused on attachment-related stress regulation and its effect on the reactivity of the hypothalamus pituitary adrenal (HPA) axis in adults using self-report measures (Heinrichs et al., 2003; Ditzen et al., 2007; Quirin et al., 2008). In addition, a few studies investigated adult attachment and the HPA-axis response during social stress (Gordon et al., 2010; Pierrehumbert et al., 2012). In these studies the interplay of oxytocin and cortisol in response to the Trier Social Stress Test (TSST) was investigated: Pierrehumbert et al. (2012) examined for the first time the interplay of attachment representation and plasma oxytocin and serum cortisol responses under stress in a mixed clinical and healthy sample (*N* = 74) using the TSST. Here, the AAI was used to determine attachment representations. Subjects with a secure attachment classification reported relatively low subjective stress; they presented a moderate response of cortisol and a high level of oxytocin after the TSST. Subjects with a dismissing classification reported moderate subjective stress; they presented an elevated cortisol response, and moderate levels of oxytocin due to social stress. Subjects with a preoccupied classification presented moderate levels of subjective stress, and of HPA response, and a relatively low level of oxytocin. Finally, participants with an unresolved classification presented a suppressed HPA axis response and moderate levels of oxytocin after TSST application (Pierrehumbert et al., 2012). However, using the TSST as a social challenge, an increase of plasma oxytocin in response to the acute social challenge test was not detectable (Ditzen et al., 2007). In this study, Ditzen et al. (2007) investigated the influence of couple interaction on cortisol and oxytocin responses to stress in women and revealed lower cortisol levels but no changes in oxytocin levels due to positive physical partner contact prior to the stress exposure (Ditzen et al., 2007). Ditzen et al. (2008) investigated the effects of the interplay of adult attachment and social support on psychological and cortisol responses to psychosocial stress using the TSST. State anxiety, mood and salivary cortisol levels were repeatedly assessed before and after stress. They found that social support alone reduced cortisol responses to stress (Ditzen et al., 2008). However, attachment seems to be a strong predictor of oxytocin and cortisol levels in the periphery, resulting in effects on state and trait anxiety. These data support the notion that attachment representations may affect stress responses and suggest a specific role of oxytocin in both the attachment and the stress system. These studies show that attachment representations are associated with characteristic oxytocin and cortisol responses in response to a social stress test.

While previous studies focused on the effects of social stress paradigms on cortisol and oxytocin responses, to our knowledge the direct effects of an attachment-related paradigm on peripheral oxytocin as well as cortisol levels before and after an attachment task have not been investigated so far. Therefore previous obtained data investigating the direct relation between attachment-related stress and peripheral oxytocin and cortisol levels seemed to be inconsistent and more research in this area is needed. According to that point, in this study we used the AAP to classify the mothers’ attachment representations. Additionally, the AAP served as a stimulus to potentially activate the attachment and caregiving system, since attachment/caregiving situations are depicted in the AAP (see “Materials and Methods” Section). Following George and Solomon (2008) model, when the caregiving system is activated and the mother becomes distressed by the situation or the child, the mother’s attachment system is also likely activated. Peripheral blood was collected to generate plasma and serum for the assessment of oxytocin and cortisol, respectively.

(i) We hypothesized an increase of oxytocin level after the AAP. (ii) We expected a possible change in the cortisol level. (iii) The investigation of attachment group differences was of exploratory nature. Nevertheless, we asumed a stronger oxytocin increase after the AAP in securely attached mothers compared to insecurely attached mothers and an increase in cortisol in the insecurely attached groups compared to the securely attached group.

## Materials and Methods

### Participants

**Figure 1:**
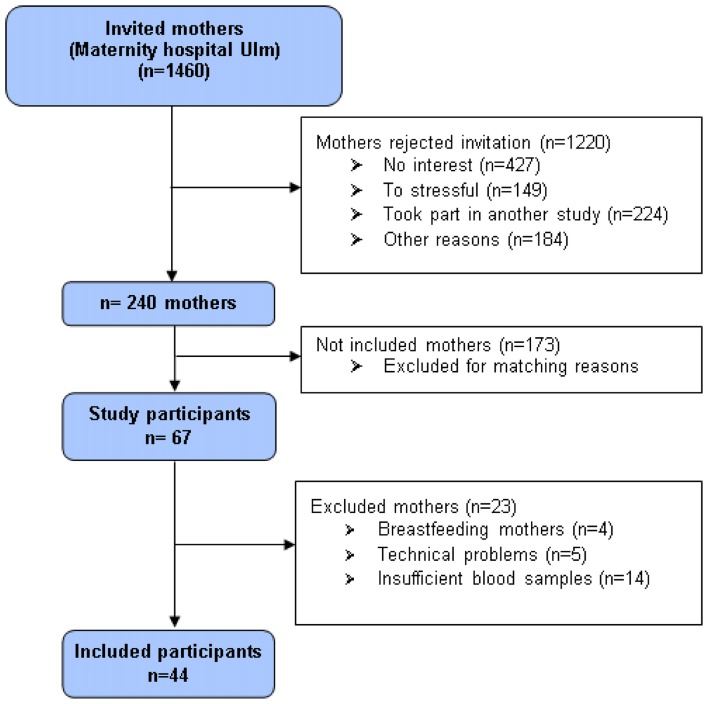
**Flowchart showing criteria of participants in- and exclusion**.

At the maternity ward of the university hospital Ulm, 1460 women who recently gave birth to a child constituted the pool of potential study participants. Exclusion criteria were age under 18, insufficient knowledge of the German language, and severe complications during parturition or severe health problems of mother or child, current drug consumption or psychotic disorders. Participants received 10 € as compensation. A total of 240 mothers provided written informed consent at *t*_0_ and were invited for data assessment 3 months postpartum (*t*_1_). As most mothers were still breastfeeding 3 months postpartum, we invited them to bring their infants along. In total, *n* = 67 mothers participated at *t*_1_. The adult attachment representation was assessed with the AAP, and peripheral blood samples were collected by venipuncture before and after the AAP. Data of four cases had to be excluded from final analyses since infants became so unsettled that mothers were needed to breastfeed them during the AAP. For five mothers, AAP narratives were missing or incomplete due to technical problems (recording device did not work, or recordings had to low-noise). Another 14 women had to be excluded due to insufficient amount of blood for radio-immuno-assay (RIA) analyses. Thus, for *t*_1_, the data of *n* = 44 mothers (age range 21.9–44.2 years, mean 33.6 years [SD 5.4 years]) was available (Figure 1). Participants received another 40 € as a compensation for the participation in *t*_1_. The study was approved by the Ethics Committee of the Ulm University. Written informed consent was obtained from all subjects in accordance with the Declaration of Helsinki.

### Attachment Measure

The Adult AAP System (George and West, 2012) assesses the attachment status in adults using a set of picture stimuli. The stimulus set includes eight line drawings, a warm-up picture and seven attachment scenes of individuals in attachment situations when they are alone or in potential attachment dyads. Participants are asked to tell a story to each picture. The stories are audio-recorded and analyses are done from verbatim transcripts. Each stimulus response is coded for attachment-related content and defensive processes. Pictures with a “alone scene” (i.e., stimuli that portray individuals alone) are evaluated for agency of self (internalized secure base, haven of safety in the context an attachment-caregiving relationship, capacity to act) and connectedness (i.e., desire and ability of the character in the story to be in a relationship defined by a behavioral system [e.g., attachment, caregiving, affiliative, sexual]). The dyadic pictures (i.e., stimuli that portray individuals in attachment-caregiving dyads) are judged by their degree of synchrony in the interactions (i.e., synchrony is evaluated based on elements of partnership or elements of mutual enjoyment). The AAP evaluates the three forms of defensive processes: deactivation (avoidance), cognitive disconnection (ambivalence), and segregated systems (attachment fear and its resolution). The AAP designates four attachment classifications based on the analysis of the coding dimensions across the entire set of seven attachment stories. Individuals with secure attachment (F) show a high level of agency, connectedness and synchrony in attachment relationships in their narratives. If they use defensive strategies, they serve a more flexible integration at the representational level (high agency, e.g., thinking processes). Individuals with insecure-dismissing or insecure-preoccupied (E) attachment are characterized by functional or absent relationships in the stories. Those with dismissing representation rather use “deactivation” (represented, e.g., by rejection, power or achievement), whereas those with a preoccupied representation use a high amount of “cognitive disconnection” as a characteristic defense (represented, e.g., by conflicts, vagueness or anger). Individuals with unresolved trauma (U) are overwhelmed by topics related to attachment-related trauma (e.g., danger, isolation, fear or threat) and loss with no indications of the character’s capacity to act, like protection from frightening and dangerous situations and no internalized available attachment figure providing comfort and security. For more complete details of the coding system and classification, see George and West (2012).

Studies provide evidence of excellent concurrent validity of the AAP with the AAI, test-retest reliability, inter-rater reliability and discriminant validity in healthy controls and clinical patients. Results from a large-scale psychometric investigation including 144 adult participants demonstrate excellent inter-judge reliability; the concordance rate for two judges on the four-group classifications were 90%, *κ* = 0.85, test-retest reliability (after 3 months 84% remained in the same attachment category; *κ* = 0.78) and discriminant validity. To evaluate the concurrent validity, AAP classifications were compared to independent AAI classifications. The concordance rates for the four-group classifications were 90%, *κ* = 0.84, and for the two groups (secure vs. insecure) even 97%, *κ* = 0.89 (George and West, 2001, 2012; Buchheim and George, 2011). All AAP protocols were analyzed by a highly experienced and reliable rater (A.B.). The personal codes of participants and time (“before” or “after” the AAP administration) were eliminated in the transcribed protocols. Hence, the judge was blind with respect to the time sequence and personal pairings.

### Design

Mothers had their last meal 2–3 h before arriving in the laboratory between 12 pm and 1 pm. Mothers were allowed to have a regular breakfast, but they were asked to fast for at least 3 h prior to study participation. They were allowed to drink water only but no coffee or tea before and during the AAP. Mothers, who breastfed their child during the study procedures were excluded from the analysis due to the known effects of breastfeeding on the oxytocin levels. After arrival, mothers were asked to first take care of their babies (e.g., taking off warm cloth or blankets), then mothers were left alone for a short period (approximately 15–20 min) to bring the endocrine levels to a baseline level. Following this short resting period the first venipuncture and blood collection was performed. Afterwards mothers were introduced to the experimental procedures. In the AAP, participants were asked to tell a story for each AAP picture: “What is happening in the scene?”, “What led up to the scene?”, “What are the characters thinking or feeling?”, “What might happen next?”. The procedure lasted about 20 min (see “AAP Duration” Section below). Missing duration values were caused by incorrect settings of voice recorders. The AAP interviews were administered by trained psychologists in a standardized manner. Immediately following the attachment task, a second blood sample was collected again via venipuncture. Mothers were allowed to hold their babies in their arms. Mothers who needed to breastfeed during the AAP or during blood collection were excluded from further analyses. In the majority of cases, infants were sleeping in their baby carriages during the experiment. However, if infants became restless, mothers were allowed to hold them in their arms. After finishing the AAP procedure and the second blood sampling, snacks and water were offered and mothers were able to take care of their babies.

### Blood Collection and Sample Preparation

Blood samples were drawn from antecubital veins into 7.5 ml vacutainer blood monovettes containing EDTA (Sarstedt, Germany) and 7.5 ml Z-Gel monovettes (Sarstedt, Germany). EDTA monovettes and tubes were ice-chilled and serum monovettes were stored at room temperature. EDTA monovettes were centrifuged at 4°C at 1.300 g for 15 min and serum monovettes were centrifuged at 4°C at 1.500 g for 10 min. Supernatants were stored at −80°C until further assay analysis. Aliquots of 250 μl were stored at −80°C until analyses.

### Determination of Oxytocin and Cortisol Levels

Oxytocin was determined by standardized RIA (RIAgnosis, Max-Planck-Institute, University of Munich). Determination of cortisol (nmol/l) was realized using a chemiluminescence-immuno-assay CLIA (IBL international—Hamburg) at the Institute of Biopsychology, University of Dresden. Samples were shipped on dry ice. All procedures were performed according to the manufacturer’s protocols.

### Statistics

The study focused on the reactivity of oxytocin and cortisol levels in response to the AAP. For the original values of these two parameters, the normality assumptions for the pre values, post values, and pre-post differences were rejected both by the exact one-sample Kolmogorov-Smirnov test with Lilliefors correction and Shapiro-Wilks test. For the logarithmic values, the normality of pre-post differences by oxytocin remained rejected by both considered normality tests (see Table 1) and the requirements allowing the application of the paired *t*-test (or mathematically equivalent GLM models) were not satisfied.

**Table 1:** **Tests of normal distribution for oxytocin and cortisol levels (*n* = 44)**.

		Kolmogorov-Smirnov test (Lilliefors correction) *p*	Shapiro-Wilk test *p*
Oxytocin level	before AAP	<0.001	<0.001
	after AAP	<0.001	<0.001
	Pre-post difference	<0.001	<0.001
Cortisol level	before AAP	0.001	0.002
	after AAP	0.008	0.003
	pre-post difference	0.025	0.036
Logarithm of oxytocin level	before AAP	≥0.200	0.726
	after AAP	≥0.200	0.903
	pre-post difference	0.003	0.022
Logarithm of cortisol level	before AAP	0.120	0.241
	after AAP	0.048	0.012
	pre-post difference	≥0.200	0.719

*Significant values indicate violations of the normality assumption*.

Other strict monotone transformations like inverse or square root did not lead to the distribution desirably satisfying the test assumption as well. Hence, we decided to apply the conservative exact sign test. This test compares the number of participants with increased and decreased parameter values. Results of the sign test are robust, because they are invariant to any strict monotone transformation. The association between increase:decrease ratios for oxytocin and cortisol were tested by the exact Fisher’s test. The correlations between oxytocin levels, cortisol levels and psychometrical scales and subscales were tested by the Spearman’s rank correlation coefficient. All these procedures are invariant to any strict monotone transformation of measured values as well. This implies that tests for originally measured and log-transformed values are mutually equal. In tables, we present means and standard deviations of originally measured hormone level values. These statistical examinations were performed by two-sided tests using the significance level *p* = 0.05. Statistical analyses were performed using the software system IBM SPSS 23.

## Results

### AAP Duration

The observed mean duration of the AAP recordings was 17.5 min (*n* = 35; time range 9–34 min; *SD* = 5.4 min). No significant Spearman’s correlation was found between the AAP duration on the one side and the pre and post values of the hormonal parameters oxytocin and cortisol on the other side (*n* = 35; oxytocin pre *r*_s_ = 0.201, *p* = 0.248; post *r*_s_ = 0.278, *p* = 0.106: cortisol pre *r*_s_ = 0.185, *p* = 0.287; post *r*_s_ = 0.013, *p* = 0.941). Furthermore, we found no significant differences in the duration of the AAP between the four different attachment representation groups (Kruskal-Wallis test, χ^2^_(3)_ = 1.213, Monte Carlo with 10^8^ simulations: *p* = 0.764).

### Attachment Representations

The four attachment representations in the study sample of *n* = 44 mothers were distributed as follows: F (secure) 9 (21%), Ds (dismissing) 16 (36%), E (preoccupied) 8 (18%), U (unresolved/disorganized) 11 (25%).

### Oxytocin Plasma and Cortisol Serum Levels

**Figure 2:**
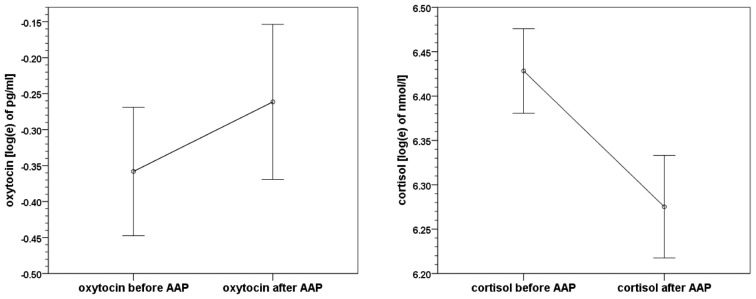
**Means and standard errors of mean of oxytocin and cortisol levels before and after the Attachment Projective Picture System (AAP) administration**.

On average, oxytocin levels increased from 0.83 pg/ml (*SD* 0.57, *SEM* 0.08) to 0.98 pg/ml (*SD* 0.75, *SEM* 0.11) from pre to post AAP. The pre and post mean values of oxytocin and standard errors of mean are shown in Figure 2. The average change of measured oxytocin levels was +0.15 pg/ml (*SD* 0.46, *SEM* 0.07); see Figure 2 and the last row in Table 2. This increase was observed in the majority of mothers (73%); the increase:decrease relation 32:12 is significant by the exact sign test (*p* = 0.004, two-sided).

**Table 2:** **Oxytocin levels before and after the Adult Attachment Projective Picture System (AAP) in the sample and four attachment groups**.

AAP	Sample size *n*	Oxytocin before *m_1_* *s_1_*	Oxytocin after *m_2_* *s_2_*	Oxytocin change *m_c_* *s_c_*	Paired effect size *d_z_*	Exact sign test increase:decrease	*p*
F-secure	9	0.60	0.70	+0.10	+0.17	3:6	0.508
		*0.27*	*0.55*	*0.63*			
Ds-dismissing	16	1.05	1.16	+0.11	+0.47	13:3	0.021
		*0.81*	*0.97*	*0.24*			
E-preoccupied	8	0.57	0.70	+0.13	+0.40	7:1	0.070
		*0.27*	*0.37*	*0.32*			
U-disorganized	11	0.89	1.16	+0.26	+0.41	9:2	0.065
		*0.34*	*0.69*	*0.64*			

Whole sample	44	0.83	0.98	+0.15	+0.33	32:12	0.004
		*0.57*	*0.76*	*0.46*			

*Oxytocin, plasma levels in pg/ml; *m*, *s*: means and standard deviations of pre, post and pre-post change values; d_z_ = m_c_/s_c_, paired effect size; *p*, two-sided exact sign test, reached significance level*.

Cortisol levels decreased from 650.2 nmol/l (*SD* 213.8, *SEM* 32.2) to 568.7 nmol/l (*SD* 211.3, *SEM* 31.9). The average change in cortisol level was −81.5 nmol/l (*SD* 212.6, *SEM* 32.1); see Figure 2 again and the last row in Table 3. A decrease of cortisol was observed by the majority of mothers (73%); the increase:decrease relation 12:32 is significant by the exact sign test (*p* = 0.004, two-sided).

**Table 3:** **Cortisol levels before and after the AAP in the sample and four attachment groups**.

AAP	Sample size *n*	Cortisol before *m_1_* *m_1_*	Cortisol after *m_2_* *m_2_*	Cortisol change effect size *m_c_* *m_c_*	Paired increase: *d_z_*	Exact sign test decrease	*p*
F-secure	9	752	510	−242	−1.12	0:9	0.004
		*255*	*147*	*126*
Ds-dismissing	16	624	558	−67	−0.40	6:10	0.454
		*178*	*147*	*167.5*
E-preoccupied	8	681	597	−83	+0.49	2:6	0.289
		*290*	*272*	*171*
U-disorganized	11	582	612	+29	+0.12	4:7	0.549
		*148*	*291*	*239.5*

Whole sample	44	650	569	−82	−0.38	12:32	0.004
		*214*	*211*	*213*

*Cortisol, serum levels in nmol/l; *m*, *s*: means and standard deviations of pre, post and pre-post change values; d_z_ = m_c_/s_c_, paired effect size; *p*, two-sided exact sign test, reached significance level*.

**Figure 3:**
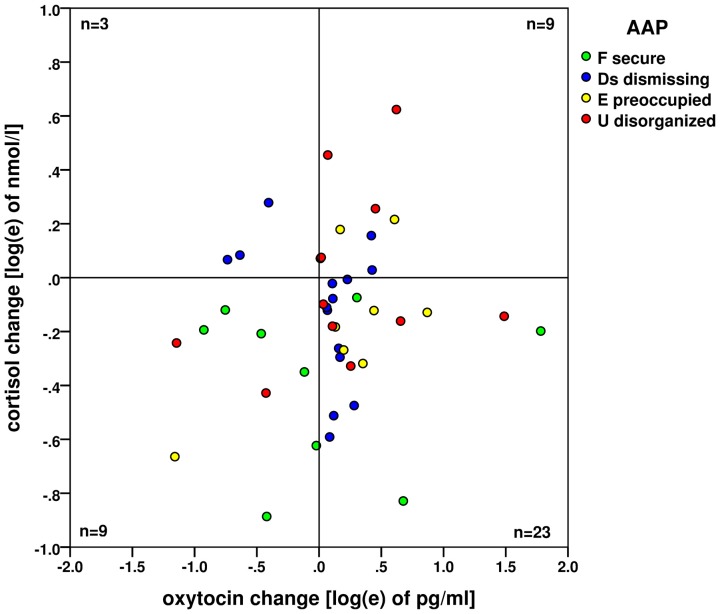
**Changes of oxytocin and cortisol levels (on the logarithm base *e* scale) before and after the AAP administration.** No association (based on the case frequencies in four quadrants) between the oxytocin increase and cortisol decrease—both processes are likely independent. Attachment types are indicated by colored dots: green: (F) secure; blue (Ds) dismissing; yellow (E) preoccupied and red (U) disorganized.

A significant increase in oxytocin levels and a significant decrease in cortisol levels were found. However, the changes were not significantly associated, see Figure 3. The concurrent oxytocin increase and cortisol decrease - shown on the logarithm scales here - were observed by the majority of 23 participants (52%), the frequencies of all four combinations of oxytocin/cortisol changes are shown. The statistics and measures based on frequencies in this fourfold 2 × 2 table (23, 9, 9, 3) did not find any association between the increase/decrease of oxytocin and cortisol (Pearson χ^2^_(1)_ = 1.213; exact two-sided Fisher’s test *p* = 0.763, Cramer’s *V* = 0.031).

### Oxytocin and Cortisol Levels in the Four Attachment Groups

The values of oxytocin and cortisol levels between the four AAP groups were compared by the non-parametric Kruskal-Wallis test, using the Monte Carlo approach with 10^7^ simulations. No significant differences between attachment groups were found for the oxytocin pre values (*p* = 0.055), oxytocin post values (*p* = 0.192), cortisol pre values (*p* = 0.537) and cortisol post values (*p* = 0.908).

Contrary to our expectation, the increase in oxytocin within the secure group was observed only in 33% cases; in all three insecure groups its proportion was over 80% (see Table 2). Comparing these proportions mutually between groups, a general significant difference between the four groups was found (exact Fisher test for 4 × 2 table: *p* = 0.046). The mentioned increase proportion in the secure group was also significantly lower than the proportion in joint insecure groups (Fisher test for 2 × 2 table: *p* = 0.007 two-sided).

According to our expectation, the decrease of the cortisol level was prevailing by securely attachment mothers (100%, see Table 3). Comparing these proportions mutually between groups, no general significant difference between the four groups was found (exact Fisher test for 4 × 2 table: *p* = 0.178). As an exploratory result not corrected for the simultaneous inference, the significantly higher decrease proportion was found in the secure group (Fisher test for 2 × 2 table: *p* = 0.047 two-sided).

## Discussion

### Oxytocin Increase, Attachment Stress and Attachment Representations

Increased oxytocin levels were observed after the AAP compared to baseline. This suggests, that the AAP is not only a valid instrument for assessing attachment representations, but may also activate a special pattern of physiological reactions in mothers, like increased oxytocin plasma levels after the AAP. Following studies of different patterns of physiological responsiveness associated with attachment groups (Dozier and Kobak, 1992), we expected different attachment representation to differ with respect to oxytocin. This expectation was not confirmed in this study: the increase in oxytocin was independent of the mother’s attachment representation, but subsequent analyses showed that the effects were mainly driven by the insecure attachment groups which showed a significantly higher increase proportion of oxytocin compared to the secure group after the AAP. Thus, contrary to our initial expectation, secure mothers did not show higher oxytocin responses compared to insecure ones. Whereas the attachment-specific stimulus AAP may lead to an increase of circulating oxytocin in mothers, studies using specific social stress stimuli, like the TSST, revealed no changes in oxytocin levels in participants (Altemus et al., 2001; Ditzen et al., 2007; Cyranowski et al., 2008). In contrast, one study found higher oxytocin secretion in healthy women and men following psychosocial stress (TSST). According to this result oxytocin may provide an important protective mediator against the health-compromising effects of a stress exposure (Engert et al., 2016). Nevertheless, these results argue for our hypothesis that oxytocin release is sensitive to an attachment-specific stimulus, whereas social stress stimuli like the TSST seemed to induce different changes in oxytocin levels, revealing recent inconsistent results. It should be kept in mind, that lactating mothers indeed have a more sensitive oxytocin-system than other study participants. The direct comparison of oxytocin levels in our study and TSST studies with healthy participants should be discussed with caution. However, oxytocin itself is known to buffer stressful outcomes like the HPA axis activity (Gimpl and Fahrenholz, 2001), activated by psychosocial stress. We did not find an increase of cortisol responses using our attachment stimulus, suggesting that the AAP was more feasible for activating the oxytocin system in this pilot study. The increase in oxytocin was observed in all four attachment groups, contrary to the hypothesis of a higher oxytocin level in secure mothers compared to insecure ones. A study of Pierrehumbert et al. (2012) investigated oxytocin levels before and after the TSST stressor in association to attachment representations. They showed a broad range of the oxytocin level changes depending on the attachment classification: securely attached participants showed higher oxytocin levels after the stressor compared to the insecurely attached groups (Pierrehumbert et al., 2012).

### Cortisol Decrease, Attachment Stress and Attachment Representations

This study found a decrease of cortisol levels after AAP administration compared to baseline. Moreover, we observed a decrease of cortisol levels after the attachment task especially in the participants with a secure attachment representation. Accordingly, the mothers classified as insecure did not show a significant cortisol decrease. These preliminary results indicate a stronger cortisol decrease in mothers with a secure attachment representation and correspond with the study of Pierrehumbert et al. (2012) who also demonstrated moderate stress reactivity in subjects with a secure attachment classification. Individuals with a secure classification revealed a low subjective stress sense and a moderate stress reactivity (Pierrehumbert et al., 2012). This study showed that attachment representations might affect stress responses independent of the used method (attachment stress or social stress). Despite the small sample size of the attachment groups, which does not allow for final conclusions, one possible explanation from an attachment perspective might be plausible: the secure group shows a flexible integration of attachment related themes in contrast to the insecure group, which was potentially reflected on a physiological level. Indeed, in individuals with secure attachment representations a high level of agency and connectedness was observed in the narratives (George and West, 2012). Thus, these individuals might have been more confident in the AAP task, while insecure individuals might have felt more stressed through the attachment task. As mentioned before we have to take into account that we have investigated lactating mothers in our study. It is already known, that the process of lactation after the birth of a child may influence the maternal oxytocin hormone system (Gimpl and Fahrenholz, 2001). Furthermore, oxytocin and cortisol can influence each other’s releasing processes, especially in stressful or angst-inducing situations (Altemus et al., 2001; Tops et al., 2007). However, we found no significant association between hormonal changes of oxytocin and cortisol in our study setting. Consequently, more research is needed to get more detailed information about the HPA axis reaction in lactating mothers during an attachment stimulus. One possible approach should be the investigation of the stress system in non-lactating mothers during an attachment stimulus, evaluating the possible effect of lactation on HPA-axis response.

### Study Limitations

The oxytocin increase after the AAP was observed in mothers 3 months postpartum. The question arises whether a similar effect would occur also at earlier or later time-points during mother-child relationship development. Clarifying these questions could generate new information about the development of mother-child attachment. In addition, due to small sample sizes of attachment groups and thus limited power, oxytocin-related differences caused by the different attachment representations might have not been detectable. This study investigated a particular sample of individuals, namely lactating mothers with newborns. It is known there are changes in maternal oxytocin responses during lactation (Salonia et al., 2005). Therefore, we excluded all breastfeeding mothers from our analyses. Furthermore, it remained unclear whether the oxytocin response may also be due to the mother-child-contact during the AAP, as mothers were allowed to be in visual or physical contact with their child during the experiments. Therefore, we cannot rule out the possibility that the mother-infant relationship might have triggered attachment-related feelings in all mothers independent of their individual attachment status. More research is needed to clarify this issue.

Blood samples were collected twice using venipuncture instead of using a venous catheter in order to avoid discomfort of the mothers due to the catheter when taking care of their babies. This approach was favored to maintain mother’s flexibility in contact with their babies during the experimental setup. Although venipuncture induces a pain stimulus and therefore may result in acute effects on circulating stress hormones, recent studies indicate that blood sampling for most laboratory tests using either direct venous puncture or peripheral venous catheter reveal comparable results (Ortells-Abuye et al., 2014). For cortisol, resting time prior to the first blood sampling may take a minimum of 30 min, which should be ameliorated in further studies. Therefore, cortisol findings in this study should interpret with caution. Additionally, controlling for duration of the AAP revealed no significant effects on time-dependent hormone levels. Our aim was to perform the pre- and postmeasurements timely as exact as possible before and after the AAP interview. Because the duration of this interview is not constant by its nature the time interval between two measurements could not be constant as well. A main limitation of the study was the lack of placebo conditions where mothers were in contact with their child without the influence of the AAP. Due to our study design this additional condition was not possible to include.

The AAP is a valid measure to assess attachment representations and its feasibility as an activating stimulus in neurobiological studies was shown in several previous studies. In this study, we were not able to differentiate whether the maternal attachment or the maternal caregiving system was activated predominantly by the AAP. Since we have examined lactating mothers and the AAP presents several stimuli depicting parent-child interaction, we supposed that both systems might have been active during the task. Further, there is only limited work that distinguished mothers’ attachment and caregiving assessments (George and Solomon, 1996, 2008). For mothers (and fathers as well), there is sometimes visible overlap between the AAP stories with their caregiving experiences. This is to be expected because the two systems mutually inform each other (George and Solomon, 2008). Activating the attachment system using the AAP, however, has been demonstrated to produce an overall representation of how children seek support from caregivers, irrespective of the overlaps. The AAP might also activate the caregiving system for many mothers, and moreover data show that the AAP is an overarching attachment system measure in different contexts (see also Isaacs et al., 2010).

Finally, the oxytocin effect could be caused by other factors than the AAP procedure; for instance, oxytocin release might have been stimulated by mother-child proximity and contact. Also the cortisol effects may be affected by other factors, like a stressful atmosphere by a crying child during the AAP or the relatively short habituation time before the basal blood collection. To answer this question, a control group of non-lactating mothers and a non-attachment related narrative task as a control condition (placebo group), are needed in future studies.

### Conclusion

This study is the first to use the AAP as an instrument with the potential to activate the attachment and/or the caregiving system in an experimental setting. Measuring stress- and attachment-related hormones our results confirmed that the AAP, indeed, led to an increase in oxytocin levels. Oxytocin increase was similar in all attachment representation groups, but was mainly driven by the insecure attachment groups who showed a significantly higher increase proportion of oxytocin compared to the secure group after the AAP. Analogously, cortisol decrease was observed over all attachment groups, and was particularly pronounced within the secure attachment representation. A more detailed investigation of the differential effects of secure and insecure attachment was limited by the small sample sizes of the attachment subgroups. When interpreting these findings it should be kept in mind that the study investigated breastfeeding mothers. Nevertheless, the study indicates that the AAP might be able to induce an increase in oxytocin, although this has to be replicated in future studies with non-lactating mothers as well as males.

## Author Contributions

This study was a pilot study to a larger BMBF-funded project on “Stress resilience in the transgenerational transmission of childhood maltreatment”. The pilot study was conceptualized by I-TK, AB, HG, CW and AK. The coding of attachment interviews were conducted by AB. The study setup and data collection were organized by KS, SK, AB, CW, CD-W, HG and AK. KS and A-LH conducted the AAP interviews. KS, A-LH, CD-W and SK controlled data entry and organized blood analyses. DP designed and performed the statistical data analyses; DP also contributed substantially to the psychological result interpretation. CW, I-TK, KS, A-LH and AK provided important intellectual contribution in commenting and revising the manuscript. SK, AB and DP wrote the manuscript and edited its final version. We would like to thank Traudl Hiller for substantial help in performing blood drawings and in the processing of blood samples.

## Conflict of Interest Statement

The authors declare that the research was conducted in the absence of any commercial or financial relationships that could be construed as a potential conflict of interest.

## Abbreviations

AAP: Adult Attachment Projective Picture System
BPD: Borderline personality disorder
Ds: dismissing
E: preoccupied
F: secure
HPA: hypothalamus pituitary adrenal
SAM: sympathetic adrenal medullary
TSST: Trier Social Stress Test
U: unresolved.
